# Music video emotion classification using slow–fast audio–video network and unsupervised feature representation

**DOI:** 10.1038/s41598-021-98856-2

**Published:** 2021-10-06

**Authors:** Yagya Raj Pandeya, Bhuwan Bhattarai, Joonwhoan Lee

**Affiliations:** Department of Computer Science and Engineering, Jeonbuk National University, Jeonju, South Korea

**Keywords:** Psychology and behaviour, Engineering

## Abstract

Affective computing has suffered by the precise annotation because the emotions are highly subjective and vague. The music video emotion is complex due to the diverse textual, acoustic, and visual information which can take the form of lyrics, singer voice, sounds from the different instruments, and visual representations. This can be one reason why there has been a limited study in this domain and no standard dataset has been produced before now. In this study, we proposed an unsupervised method for music video emotion analysis using music video contents on the Internet. We also produced a labelled dataset and compared the supervised and unsupervised methods for emotion classification. The music and video information are processed through a multimodal architecture with audio–video information exchange and boosting method. The general 2D and 3D convolution networks compared with the slow–fast network with filter and channel separable convolution in multimodal architecture. Several supervised and unsupervised networks were trained in an end-to-end manner and results were evaluated using various evaluation metrics. The proposed method used a large dataset for unsupervised emotion classification and interpreted the results quantitatively and qualitatively in the music video that had never been applied in the past. The result shows a large increment in classification score using unsupervised features and information sharing techniques on audio and video network. Our best classifier attained 77% accuracy, an f1-score of 0.77, and an area under the curve score of 0.94 with minimum computational cost.

## Introduction

Human history shows the importance of music in human life^1,2^, and the importance has increased in the digital age. Life without music is hard to imagine because it is deeply associated with our society, culture, and mental satisfaction. Music is a source of pleasure which has psychological benefits including stress management, and improved mood and motivation. Some studies^3–5^ have investigated the question “why do we listen to music?” and found that music boosts our mood, feeling, and self-confidence. Additionally, music is an extremely safe, non-invasive, easily accessible, and non-expensive media.

A music video is a commercial product containing music with imagery to promote a song or album. Modern music videos use a wide range of filmmaking styles that are used as a marketing tool to promote the sale of music recordings. Many music videos present specific images and scenes from the song's lyrics, while others take a more thematic approach. To produce a music video, the director tries to create a visual depiction that corresponds to their subjective interpretation of the emotion expressed in a piece of music.

In the digital age, social media and music streaming services have increased the demand for music videos. Most music video retailers sell emotion as a piece of music via online and offline businesses. Industrially, music emotion analysis has been used for personalized and generalized music recommendation; emotion-based music searches; automatic music creation; and more. Other applications of music’s affective computing are mental health treatment, the gaming industry, advertisement, and broadcast stations.

The major challenge in music’s affective computing is data annotation because emotion has an inherent relationship derived from multiple aspects of music which vary over time. Emotion description is inherently subjective because the context of music corresponds to the associated emotion. The presentation of emotions also varies across cultural groups, languages, and intentions of a music maker. Music emotion itself is a complex representation that combines the vocalist’s emotion, the melody, and linguistic information. Music videos contain further complexity because visual and acoustic information have distinct emotion representation schemes. One possible approach to address this complexity when predicting emotion in a music video is to separately analyze the audio and visual information, and then integrate the results.

In this paper, we propose an unsupervised multimodal method for music video emotion classification. An autoencoder network with music and video information was trained on music video samples collected from the Internet without any label. Music and video multimodal network architectures were trained on more than 0.13 million music video samples for joint information representation. Initially, general 3D convolutions were used in our multimodal architecture that drastically increases the network parameters. The system complexity was reduced by introducing channel and filter separable convolution. Similarly, the complexity in the music networks using a 2D square filter (${\text{n}} \times {\text{n}}$) was minimized using rectangular temporal ($1 \times {\text{n}}$) and spectral (${\text{n}} \times 1$) filters and channel separable convolution, where n is the convolution filter size for one dimension. A multimodal transfer module (MMTM)^6^ was used in the proposed architecture for run-time information sharing and boosting. Finally, unsupervised networks were fine-tuned with an upgraded version of the music video emotion dataset of^7^. The key contributions of this study are listed below:

1. We propose an unsupervised multimodal architecture for music video feature representation using a large unlabeled dataset. Further, we fine-tuned the multimodal with a supervised music video emotion dataset of six emotion categories.
2. The complexity of the neural networks using conventional 2D/3D convolution was reduced using the channel and filter separable convolution. The proposed convolution minimized the training parameters and even improved the performance. The light weighted representation allows us to train the autoencoder of music and video using an end-to-end manner.
3. The slow–fast network^8^ strategy was applied for unsupervised music video emotion representation with MMTM module to boosts and shares the learned information of audio and video at the training time of the network.

The result shows a large increase in performance with a great reduction in parameters using the separable convolution. The unsupervised feature and information sharing and boosting methods were beneficial for affective computing of the music video contents. We compared the system performance using various evaluation metrics and visual analyzers. The result shows better qualitative and quantitative results compared to past research.

## Related work

Deep learning technology gaining popularity in recent years making our daily lives easier in a variety of ways. Aside from visual image and videos, numerous success stories in animal sound^9–12^ and the music information retrieval area^13^ have quickly spread. After the development of deep learning technology, affective computing in multimedia content has gained a lot of interest during the last 5 years. Since there are few studies of music videos, you will compare and contrast them detail in this section.

Many supervised automatic music emotion classifiers have been proposed in which manual annotation guides the system^14–18^; but the emotion annotation is a relatively costly and complex task. In contrast, an unsupervised approach does not require hard annotation. The system automatically learns from input data characteristics. If a neural network is trained on a large unlabeled dataset of music, the system automatically builds a generality to distinguish musical components. However, only a few unsupervised methods have been proposed for music emotion classification^19–21^.

As far as we know, no research has been done on video emotion analysis in the context of music video content. We'll go through some of the supervised and unsupervised video processing techniques that have been created. Our model is based on some prior arts that were used in video analysis. Supervised video classification is widely used in computer vision^22–25^. Several studies^26–30^ illustrate their results of emotion analysis of video. Recent studies explore to deal with the spatiotemporal information of video^6,31,32^. The author in^31^ proposed a system for memory-efficient video classification using cluster and aggregate models. Facial expression recognition (FER) is a sub-domain of human affective computing that uses face geometry and textual input to recognize human emotion. The majority of the studies in FER^33–35^ relied on facial context information and dominant facial landmarks. The quality of the face image, the camera distance, and the depiction of facial landmarks are the most important tuning parameters for FER. Yagya et al.^36^ proposed a supervised technique for music video emotion analysis that incorporates audio, video, and facial data. The study investigates the importance of musical elements for affective computing in music videos, and the quality of the face image is important to determine the overall music video emotion. SmallBigNet^32^ presented a method for dealing with the various perspectives in video, in which a big view directs a small view branch in 3D feature space. The works in the video domain that were discussed were only for supervised classification. Research has also been conducted for unsupervised video classification^37–39^ using convolution and recurrent neural networks. Nomiya et al.^40^ proposed unsupervised emotion classification in video using the Gaussian mixture model (GMM).

Many supervised studies have used hand-crafted spatiotemporal features based on optical flow to capture the motion information of video. This method typically includes histograms of flow^41^, motion boundary histograms^42^, and trajectories^43^ for action recognition in video data. However, it is methodologically unsatisfactory given that optical flow is a hand-designed representation, and two-stream methods are often not learned end-to-end jointly with the flow. The Slow–fast network in^6^ is based on the idea of preserving the spatial and temporal information of video using end-to-end learning. The slow branch captures static but semantically meaningful features whereas the fast branch captures the temporal information of the video sequence. Xiao et al.^44^ demonstrated that a slow–fast video network with audio improves the video action classification and detection task, but they only evaluated audio on the fast pathway. In this study, we extend this concept with audio and video in both slow and fast path and the learned information of both branches is boosted and shared using the MMTM module. The MMTM shares the information from two modalities and boosts the information using the squeeze and excitation^45^ method.

In the case of music video emotion analysis, supervised methods have been conducted using the conventional approach^46,47^ and deep learning approach^5^. At the time of writing, the authors are not aware of any unsupervised methods for music video emotion classification. We provide an unsupervised music video representation approach as well as an automatic music video emotion classification method in this work.

### Music video emotion dataset

#### Unlabeled dataset

A large dataset with precise annotation has become increasingly important for training the huge number of parameters of a data-hungry deep learning model. However, the data annotation process is relatively difficult and costly in the field of affective computing. For music videos in particular, the task is more challenging because it includes multiple information sources with their own emotion representation paradigm. The annotation on music videos is challenging even for an expert because emotion itself is a subjective task, dynamic across time, requires different emotion representation schemes for individual music video components (lyrics, music, video), and is influenced by culture. Therefore, data scarcity appears if the affective computing algorithms are data driven. The unsupervised representation helps in the data-driven method to minimize data scarcity and annotation difficulties. The unsupervised network can use a massive amount of raw data floating around the Internet and expanding day by day. The classification of human emotions included in a music videos without annotation is most economic approach in the case like music video where the labeling task is expensive and complicated.

To train the unsupervised neural network, we collected a large number of music videos from YouTube. A 30-s video clip was selected from each music video. The video clips were cut from a random temporal location of full length music video and downloaded using an automated python code. We set a search key related to music videos and downloaded both official and user-generated music videos. We filtered the clips because some were not related to music video and some had only music (not visual dynamics). The data was filtered to exclude reviews, interviews, and other music-related speech. Finally, our sample contained 0.13 million music videos for unsupervised training. The collected data were further processed using audio noise reduction techniques.

#### Labelled dataset

Several datasets have previously been proposed for supervised music emotion analysis. Some datasets^48–50^ follow the categorical model^51^, providing several discrete categories of emotions, and other datasets^14,52,53^ use the dimensional model^54^ to represent emotion as a value in 2D valence and arousal space. Similarly, like music, video emotion datasets^55,56^ have been proposed using the categorical model and others^57,58^ have used dimensional models.

DEAP dataset^59^ was the first music video dataset available with 120 data samples taken from western music. The dimensional emotion representation model was used to rate affective tags in terms of arousal, valence, and dominance, using value ranges from 1 to 9. The dataset does not include enough samples to adopt the data-driven algorithms like deep neural networks. To overcome the data scarcity problem, we prepared a music video emotion dataset using the contribution of four annotators. The dataset is an extension of^5^ with the same emotion representation framework of six emotion categories, namely: Excited, Fear, Neutral, Relaxation, Sad, and Tension. The modified music video dataset included training, validation, and test samples of 4788, 655, and 300 video samples respectively. The test samples were equally distributed over the six class categories for a fair comparison. This new music video emotion dataset (MVED) has been used in^36^ and made publicly available.

We categorized the dataset into six distinct classes based on their corresponding emotional adjectives. The “Excited” class usually includes positive emotions. The visual elements of the “Excited” class includes images of a smile on a face, movement of arms, dancing scenes, high lighting, and coloring effects. The audio components of this class include high pitch, large pitch variation, uniform harmony, high volume, low spectral repetition, and diverse articulations, ornamentation, and vibrato. The visual features of the “Fear” class reflect negative emotions via a dark background, unusual appearance, wide eyes, open mouth, a visible pulse on the neck, elbows directed inward, and crossed arms. Common visual elements in the “Tension” class are fast-changing visual scenes; crowded scenes; people facing each other; aggressive facial expressions with large eyes and open mouths; and fast limb movement. The audio elements in the “Tension” and “Fear” classes include high pitch, high tempo, and high rhythmic variation, high volume, and a dissonant complex harmony. The visual elements in the “Sad” class are closed arms, a face buried in one’s hands, hands touching the head, tears in eyes, a single person in a scene, a dark background, and slow-changing scenes. The “Relaxation” class includes ethnic music and is visually represented with natural scenes in slow motion, and single-person performances with musical instruments. The acoustic components of the “Sad” and “Relaxation” class include slow tempo, uniform harmonics, soft music, and low volume. The “Natural” class includes mixed characteristics from all the other five classes. The data samples in each class are diverse in terms of musical group, culture, nationality, language, number of music sources in one audio, and mood.

#### Data preprocessing

The raw music video data needed to be processed in an acceptable form for the neural network. Our dataset processing follows several steps for each individual data sample of music and video. The music network is trained on the real (magnitude) and imaginary (phase angle) components of the log magnitude spectrogram. The magnitude of log Mel spectrogram was kept in one channel and the phase angle representation was placed on another channel to preserve both the magnitude and phase information of the acoustic signal. The raw music video data needed to be processed in an acceptable form for the neural network. Our dataset processing followed several steps for each data sample of music and video. The music network is trained on the real (magnitude) and imaginary (phase angle) components of the log magnitude spectrogram. The magnitude of log Mel spectrogram was kept in one channel and the phase angle representation was placed on another channel to preserve both the magnitude and phase information of the acoustic signal. Several studies^60–62^ have demonstrated that phase information improves the performance of both speech and music processing.

For this work, the 30-s audio waveform x_i_ was converted to mono and then subsampled with a window size of 2048, a sampling rate of 22,050 Hz, and a time shift perimeter of 512 samples. The sampling rate varied for the slow–fast network where x_i_ in the slow path had a sampling rate of 32 kHz and the x_i_ in the fast path had a sampling rate of 8 kHz. The Fast Fourier Transform (FFT) was then applied for each window to transform the x_i_ from time domain to a time–frequency (T–F) representation X_i_(t, f). From the entire frequency spectrum, 128 non-linear Mel-scale were selected that matched the human auditory system. The use of the log Mel spectrogram has two benefit compared to wave form audio. First; it reduces the amount of data that need to process by the neural network and second; it is related to human auditory perception and instrument frequency range^63^.

**Figure 1:**
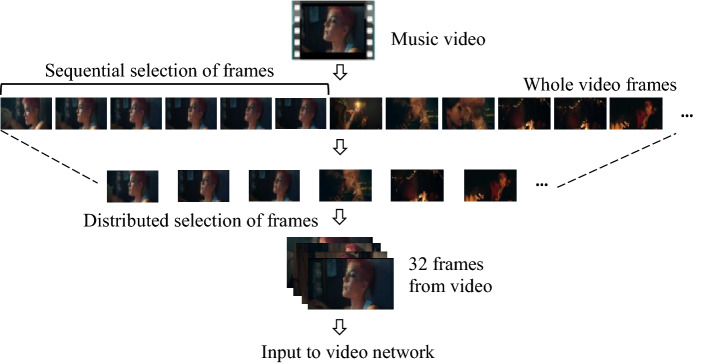
Input video processing using distributed selection of frames. The video frames are from the first 30 s of the music video “Without Me” (https://www.youtube.com/watch?v=ZAfAud_M_mg).

The sequence of images on the video were collected in a distributed manner to preserve the temporal information of the entire video sequence. Each video was converted into several frames sequences V_i_ = {v${\uptau }$, v_2_${\uptau }$, v_3_${\uptau }$, …, v_n_${\uptau }$}, where ${\uptau }$ represents equal time intervals in the video sequence. For each sample, ${\uptau }$ changes according to the total n number of fames that were extracted, as shown in Fig. 1. For the video network, 64 frames were taken in a distributed fashion. Video data was processed in a similar way to the audio data by varying the frame rate in the slow (8 frames) and fast (64 frames) branches of the slow–fast network.

After the preprocessing, the input to the audio network was A_N_ = $\sum\nolimits_{{{\text{i}} = 0}}^{{\text{N}}} {{\text{X}}_{{\text{i}}} }$ and to the video network, V_N_ = $\sum\nolimits_{{{\text{i}} = 0}}^{{\text{N}}} {{\text{V}}_{{\text{i}}} }$, where N is the data used in one batch. The multimodal input was the integrated form of the audio and video input.

### Proposed network

In this paper, we present multimodal learning using the unsupervised method. In multimodal representation, the complementary information provided by the different modalities is integrated to enhance the system capability. An autoencoder network is developed to represent the multimodal representation using audio and video information. An autoencoder is a generative model, which usually uses a latent representation bottleneck and use it to reconstruct input^64^. The CNN-based autoencoder is one of the popular unsupervised feature representation method^65,66^. At the time of writing, we are unaware of any unsupervised music or music video emotion representation method using deep learning technology. In this study, an autoencoder was trained on the encoder-decoder paradigm, where the encoder network used two multimodal architectures with a dense residual block with a variety of convolutions. The decoder network is made simple and lightweight using 2D/3D convolution. We will discuss the detail encoder and decoder network after the various convolution filter used in this study.

#### Convolution filter

**Figure 2:**
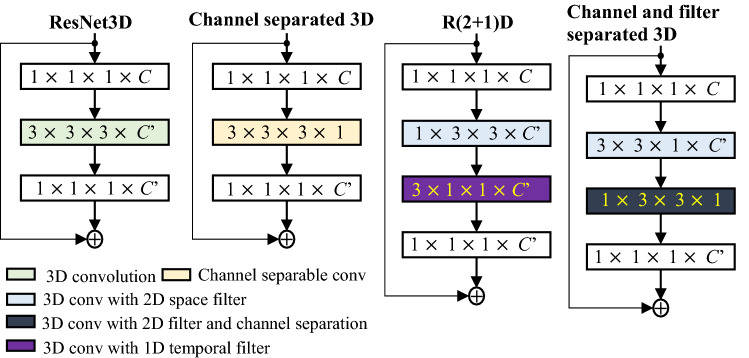
3D convolution and its variants in residual block representation.

In video processing, 3D convolution has been found to better capture spatial and motion information, but it exponentially increases the complexity of a system. Some popular 3D networks^67,68^ have included this complexity and, as a result, require a large dataset for successful training. In this paper, the complexity of 3D convolution was reduced using filer and channel separable convolution. The proposed convolution was an integrated form of channel separable convolution^69^ and (2 + 1)D convolution^70^. For the separable filter, the 3D convolution filter of size ${\text{n}} \times {\text{n }} \times {\text{n}}$ was divided into 2D space as $1 \times {\text{n }} \times {\text{n}}$ and ${\text{n}} \times {\text{n }} \times 1$, where n is the convolution filter size for one dimension. Separable filer and channel convolution was also used for the 2D audio network. The square filter of 2D convolution was divided into a temporal filter ($1{ } \times {\text{n}}$) and spatial filter (${\text{n }} \times 1$), as in^71^. The channel size was reduced to one in the sequential block of the dense residual network for the separable channel. A detailed representation of the proposed filter and channel separable convolution is illustrated in the right most configuration in Fig. 2.

#### Encoder network

Two network architectures were used for information encoding, namely the “music-video encoder network” and “separable slow–fast encoder network”. Each network included an MMTM block for information sharing and feature enhancement. The music-video network used conventional 2D/3D convolution, whereas the separable slow–fast network used filter and channel separable convolution.

**Figure 3:**
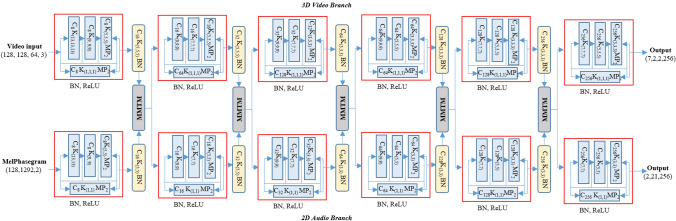
The music-video encoder network. Video stream (upper) and audio stream (lower) are connected with the MMTM information fusion block.

The music-video encoder network has two parallel branches for music and video processing with an MMTM for information exchange after each dense residual block, as shown in Fig. 3. The music network input is a two-channel time–frequency representation of audio, where the first and second channel are real and imaginary part of the sinusoidal audio signal. The spectral representation has 128 frequency bin and 1292 temporal length. Two-dimensional convolutional filters are used in each dense residual block. In each convolutional block, a batch normalization and rectified linear unit (ReLU) activation function is used for stable training. In the case of the video network, three-dimensional convolutional filters are used to capture both the spatial and temporal features of the video sequence. After each dense residual block, the output is smoothed using a convolution layer with batch normalization.

**Figure 4:**
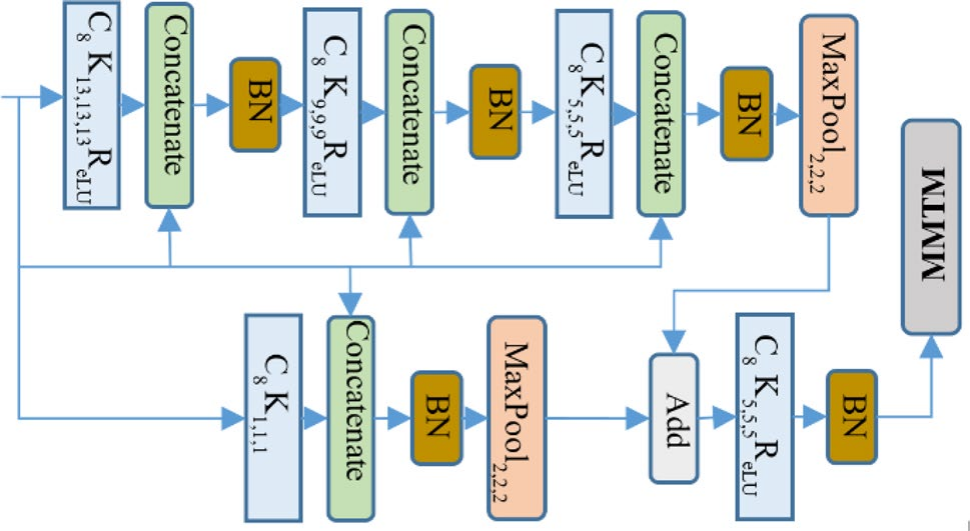
Detailed overview of the dense residual block of audio and video network (the value related to first block of video network). The symbol ‘C’ indicates the convolutional, ‘K’ indicates kernel size, ‘BN’ indicates batch normalization, ‘R_eLU_’ indicates the rectified linear unit and MMTM indicates multimodal transfer module for multimodal information fusion. The lower case value indicates the given value to corresponding symbol.

Inside each dense residual block, the max-pooling operation is used for dimension reduction in the two parallel branches. Each parallel branch is then added together and the result is smoothed with a convolutional layer. Finally, the output from each individual dense residual block of audio and video branch is passed to the MMTM block for information fusion, as shown in Fig. 4.

**Figure 5:**
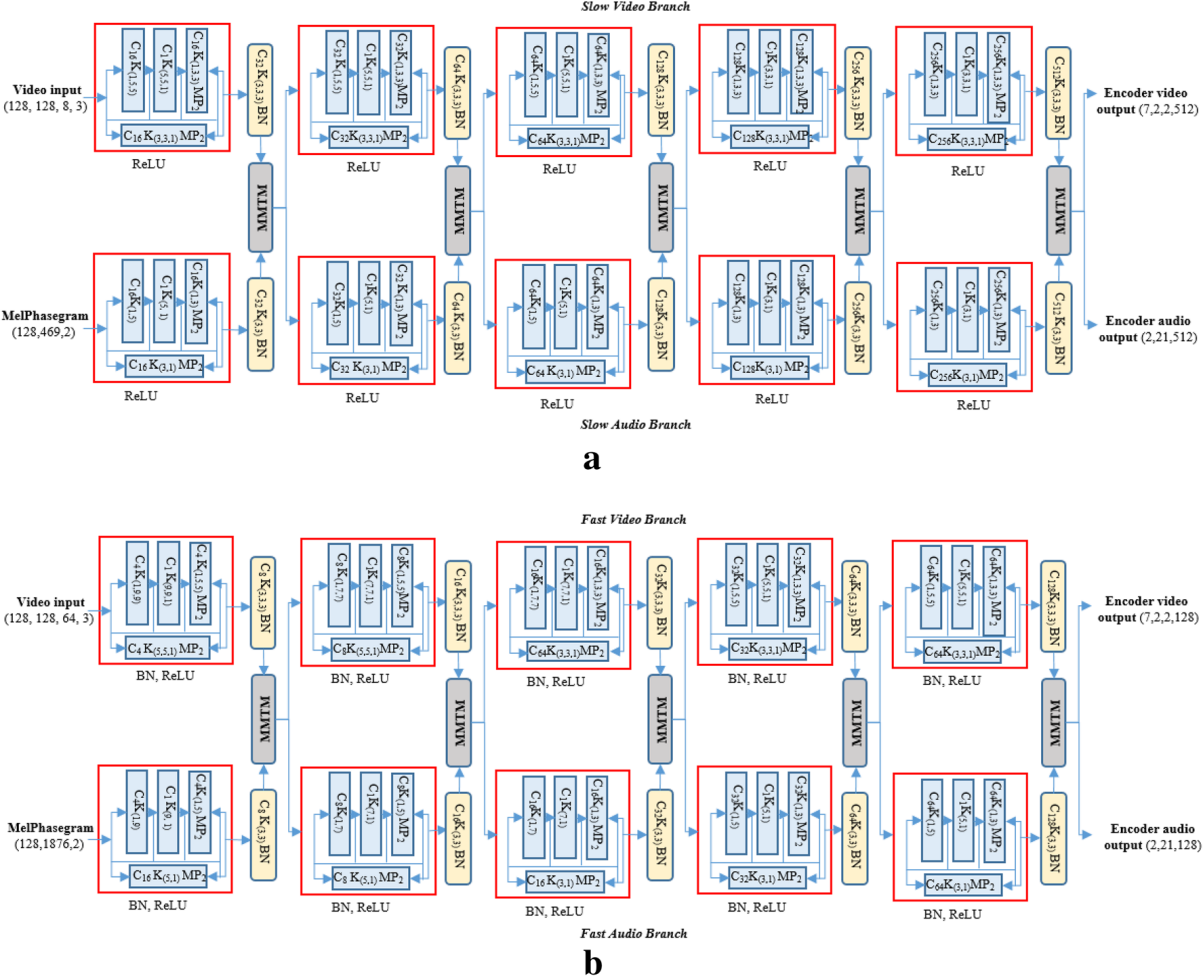
(**a**) Slow branch of the separable slow–fast music video network. (**b**) Fast branch of the separable slow–fast music video network.

The 2D/3D convolution in the music-video network increases the complexity and it is impossible to add more audio or video branches for end-to-end training. We drastically reduced the network complexity using filter and channel separable convolution and trained a slow–fast encoder network with audio and video information. Slow–fast networks can be described as a single stream to capture both spatial and motion information. The slow path is designed to capture more static but semantic-rich information, whereas the fast path is tasked with capturing fast motion. We used slow–fast representation for both the audio and video networks with because both media are spatially meaningful over time. Both slow and fast network branches are trained in parallel with information sharing using MMTM after each dense residual block. The MMTM module helps to modulate the audio and video correspondence over time. The architectural detail of the slow music video emotion network is shown in Fig. 5a. The wave audio is sampled at 8 kHz for the slow path of audio network input of size (128, 469, 2), where one channel is the log Mel spectrogram and the other is the phase angle. The slow video path includes 8 image frames with an RGB color channel.

The fast branch of the slow–fast network has a similar network structure as the slow branch. However, video frames (64 frames) and sampling rate (32 kHz) are four times larger than the slow branch. Another difference is the size of the filter in the convolutional layer inside each dense residual block. To capture the motion information of the video, we process the input with a large filter size and low feature dimension. The architectural detail of the fast branch of the network is shown in Fig. 5b.

**Figure 6:**
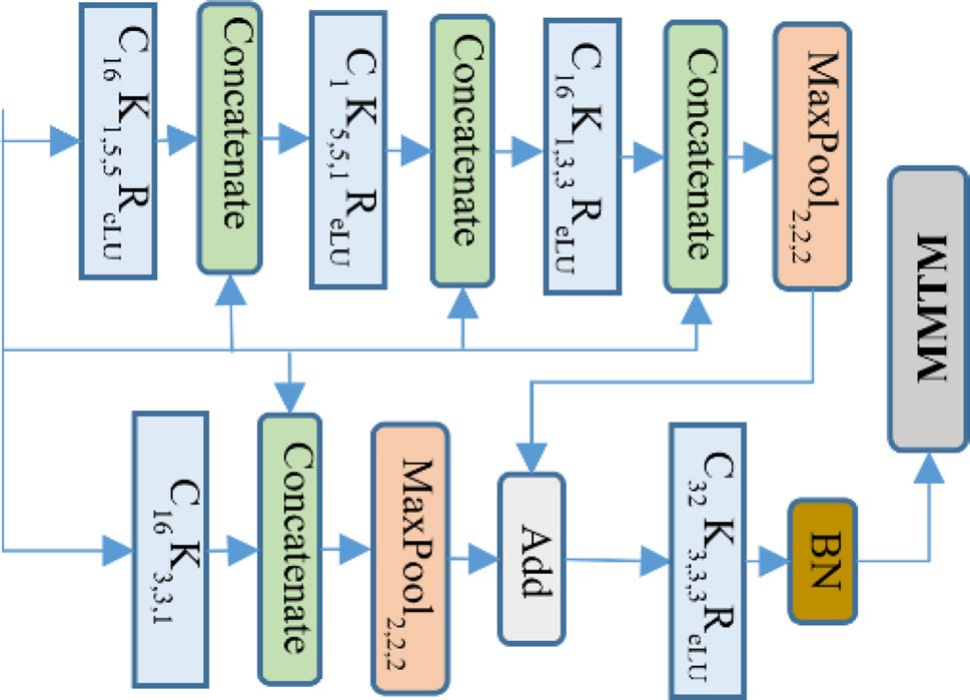
Detailed overview of the dense residual block of the audio and video slow–fast network (the first block of video network is illustrated here). The symbol description is similar as in Fig. 4.

In both fast and slow branches, the 3D convolution is limited to only one layer after a dense residual block for smoothing the learnt features. The dense residual block uses only a 2D convolutional layer with dense connection to the input so the network parameters are relatively low and easy to train in an end-to-end fashion. The detailed view of each dense residual block with filter and channel separable convolution is shown in Fig. 6.

#### Decoder network

In an autoencoder, the latent representation of input is reconstructed as output using a decoder network. A decoder network generally has the same structure as an encoder in reverse order from latent representation to input but other structures are possible. The goal of the decoder network is the reconstruction of encoded data in the original input form. The two decoder networks in this study: the “music-video decoder network” and “separable slow–fast network”, are designed to be simple and lightweight to reduce the number of network parameters of the multimodal architecture.

**Figure 7:**
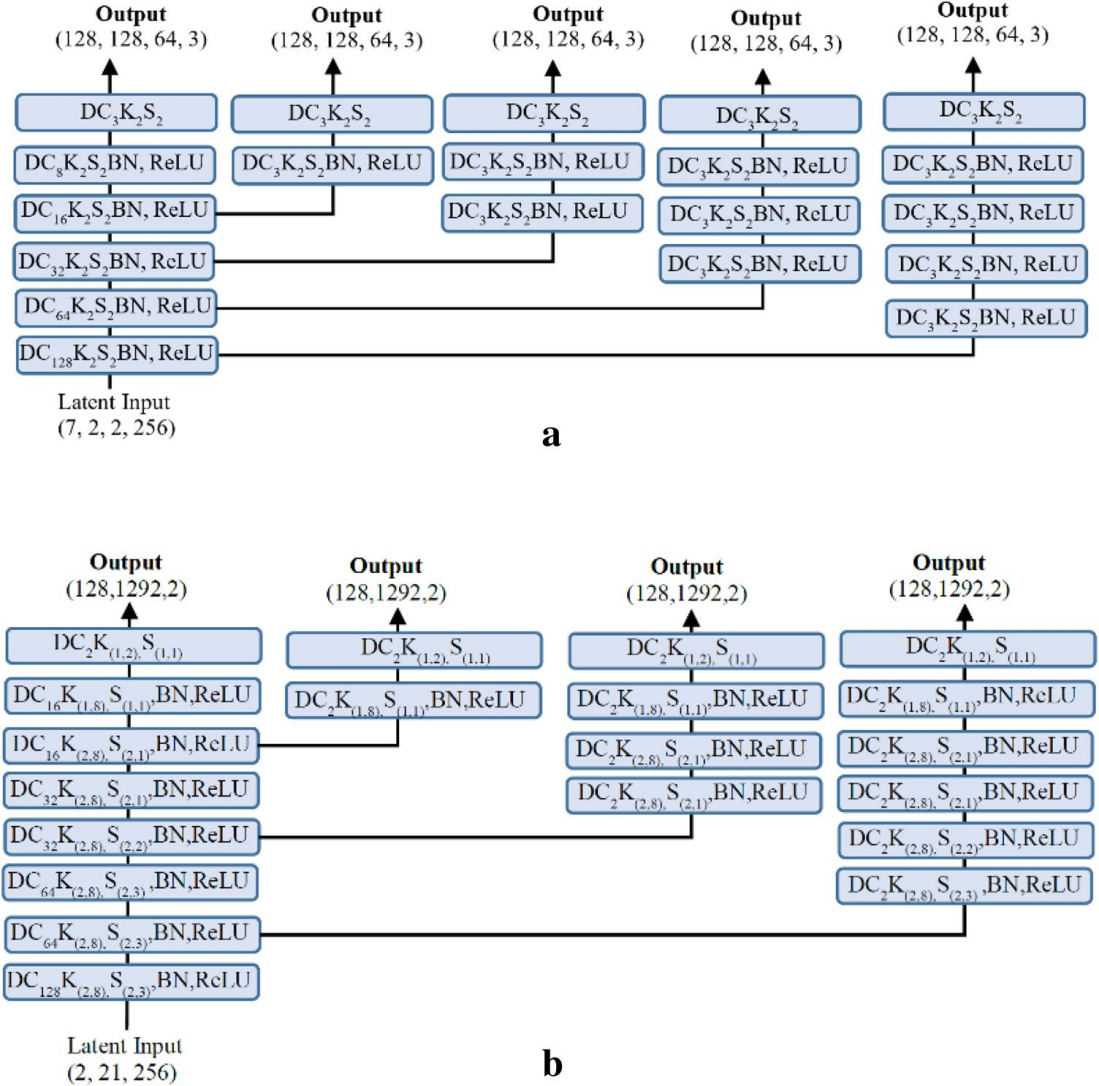
(**a**) The 3D video decoder network with multi-stage loss. (**b**) The 2D audio decoder network with multi-stage loss.

The music-video decoder network is designed to reconstruct the audio and video sequence from the separate branches. A simple 2D/3D transpose convolutional layer with batch normalization and ReLU non-linear activation function is used to upsample the feature from the latent space. The kernel size and strides are adjusted separately for each transpose convolution layer to make the final feature size equal to the input. The final video size is the same as the input, with three RBG channels, and the audio size is the same as the input log Mel spectrogram with phase information. The proposed decoder network introduces four auxiliary outputs for the video network in the expanding pathway and three auxiliary outputs for the audio network in the expanding pathway with the intention of improving the gradient propagation and decreasing the probabilities of a vanishing gradient for the deep audio–video multimodal networks. The multiple auxiliary outputs work as a kind of deep supervision and minimize the overall loss functions^72,73^. The detailed architecture of the video and audio decoder network with multiple auxiliary outputs is shown in Fig. 7a, b respectively.

**Figure 8:**
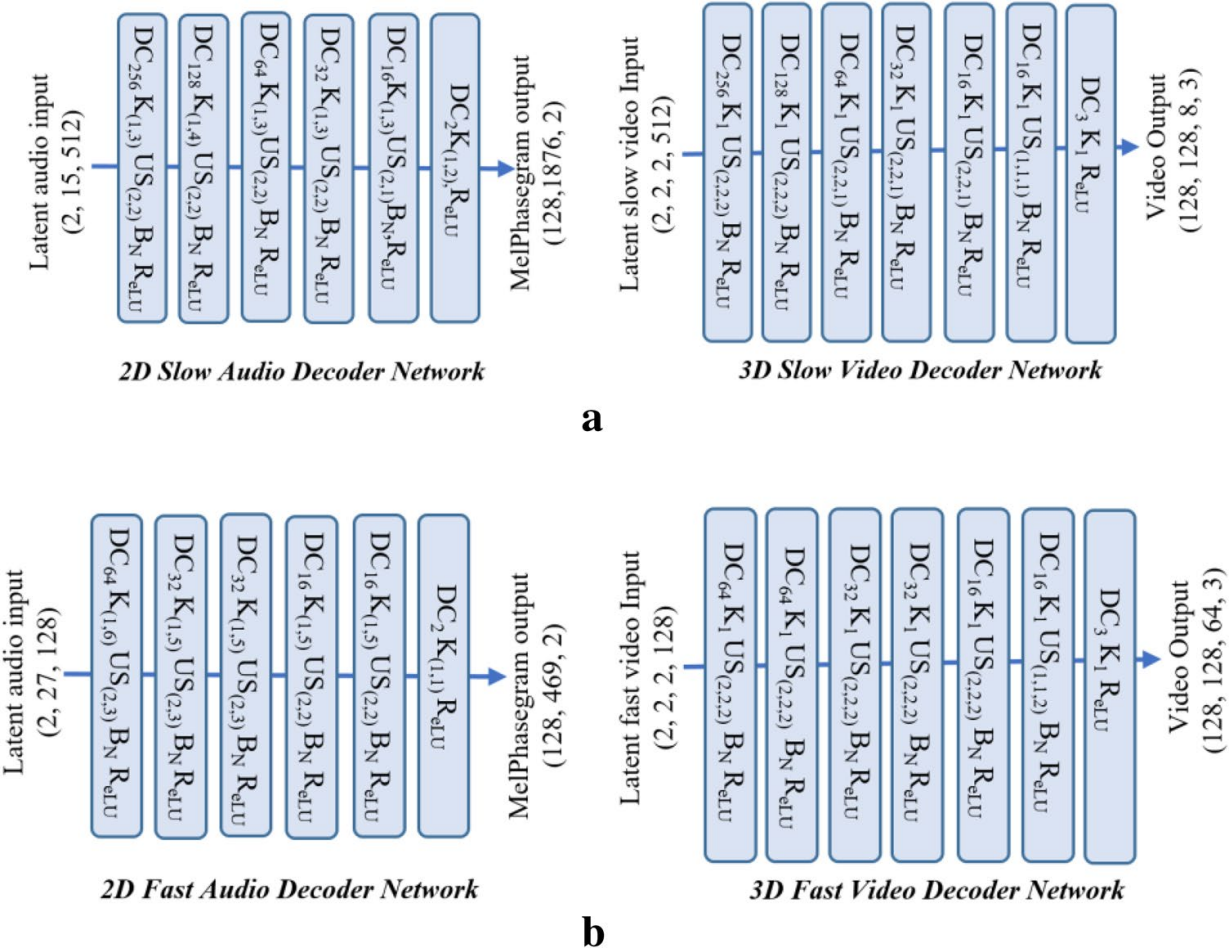
(**a**) The slow branch of the separable slow–fast decoder network. (**b**) The fast branch of the separable slow–fast decoder network.

The separable slow–fast decoder network has two audio network branches and two video network branches without information sharing (MMTM block) across the audio and video network. The decoder architecture for the audio and video network in each slow and fast branch reconstructs their respective input dimension from their latent representation. The dimension of latent space and output of each audio and video decoder for the slow and fast path is shown in Fig. 8a, b. respectively.

#### Fine-tune with labelled dataset

The unsupervised features of music video emotion can be useful in the initial phase of training, but human performance data is required for truly reliable evaluation. To exploit our unsupervised networks for real world emotion analysis of music videos, the network was fine-tuned with the labelled data using the six emotion categories. The goal of fine tuning the unsupervised feature is to identify the emotion classes to which the music video belongs. Given a fixed set of m classes c_1_, c_2_, c_3_, …, c_m_ ∈ C, audio A_N_ = $\sum\nolimits_{{{\text{i}} = 0}}^{{\text{N}}} {{\text{X}}_{{\text{i}}} }$ and video V_N_ = $\sum\nolimits_{{{\text{i}} = 0}}^{{\text{N}}} {{\text{V}}_{{\text{i}}} }$ data with batch size N, we are interested in predicting the probability P{c_i_ |(A, V)} for each of the m classes. This probability can be parameterized using the proposed multimodal M which looks at the joint representation of log Mel-spectrogram of audio and video frames in the music video to predict: P{c_i_|(A, V)} = M{(Xi(t, f)), (v${\uptau }$, v_2_${\uptau }$, v_3_${\uptau }$, …, v_n_${\uptau }$)}.

The encoder network was fine-tuned with additional global average pooling and a Softmax layer at the end. We used only five dense residual blocks of music-video encoder network for the supervised training. The features from the audio and video networks are concatenated after global average pooling. Finally, the predicted probability is passed through a Softmax activation function that pushes most probable result closer to 1 while others are pushed closer to 0.

## Results

We used large unlabeled dataset for end-to-end auto-encoder training. In this process, the network tries to discover the correlated information of the multiple music video samples and learns the relevant characteristics. We trained the unsupervised network for more than 1 month and fine-tuned the representation with the labelled data to adopt the new distribution of categories. The evaluation metrics used in this experiment are accuracy, F1-score, and the area under the receiver operating characteristic curve (ROC-AUC) scores. We visually interpreted the system performance using confusion metrics and the ROC curve. The dynamic perceived emotion was evaluated by computing the time-synchronized emotion of the music videos.

The loss function used for unsupervised feature representation was the mean squared error (MSE). MSE loss calculates the square of the difference between actual ground truth and network predicted result and average it over all the training data. The unsupervised features were fine-tuned with labelled data of music video emotion. At the decision level, all the branch information is globally aggregated and computed for a class-wise probability. The Softmax function was used for supervised training in the final layer of the neural network, which maps the output nodes in a probability value range between 0 and 1. We use the categorical cross-entropy loss function for a one-hot vector target. This function computes the binary cross-entropy for each class separately and then sums them up for the complete loss.

In this experiment, we compared the two proposed networks using the evaluation score and network complexity. The networks were compared with the result of the end-to-end training and the unsupervised representation. In both cases, as shown in Tables 1 and 2, the separable slow–fast network outperformed the music-video network. The music-video network using conventional 2D and 3D convolution was relatively complex and took more training time. The slow–fast network used the separable filter and channel convolution, and this drastically reduced the number of network parameters and increased the system performance. The MMTM block boosted the system performance up to six percent on the separable slow–fast network and also improved the result of the music-video network. The comparison of evaluation scores with or without the MMMTM block is illustrated in Table 1, where all the networks were trained on the dataset in an end-to-end manner.

**Table 1:** Effect of the MMTM block using music video test samples (training from the scratch).

Model	Test accuracy	F1-score	ROC AUC score	Parameters
Music-video network (with MMTM)	0.6800	0.68	0.915	23,078,854
Music-video network (without MMTM)	0.6600	0.64	0.897	23,034,214
Separable slow–fast network (with MMTM)	0.70333	0.70	0.919	17,118,864
Separable slow–fast network (without MMTM)	0.63666	0.63	0.899	16,930,880

**Table 2:** Music and video score on test sample after finetuning the unsupervised representation.

Model	Test accuracy	F1-score	ROC AUC score	Parameters
Music-video network (without MMTM)	0.7033	0.70	0.920	23,078,854
Separable slow–fast network (without MMTM)	0.7700	0.77	0.940	17,118,864

The unsupervised feature of using the separable slow–fast network improved classification accuracy by approximately seven percentages. The music-video network also improved with the unsupervised features, as illustrated in Table 2. The MMTM block was found to be essential in the Auto-encoder network with a small increment in network complexity. In both proposed networks, audio–video information was synchronized well using MMTM. The unsupervised feature and MMTM increased the overall performance.

### Analysis based on visual predictions

**Figure 9:**
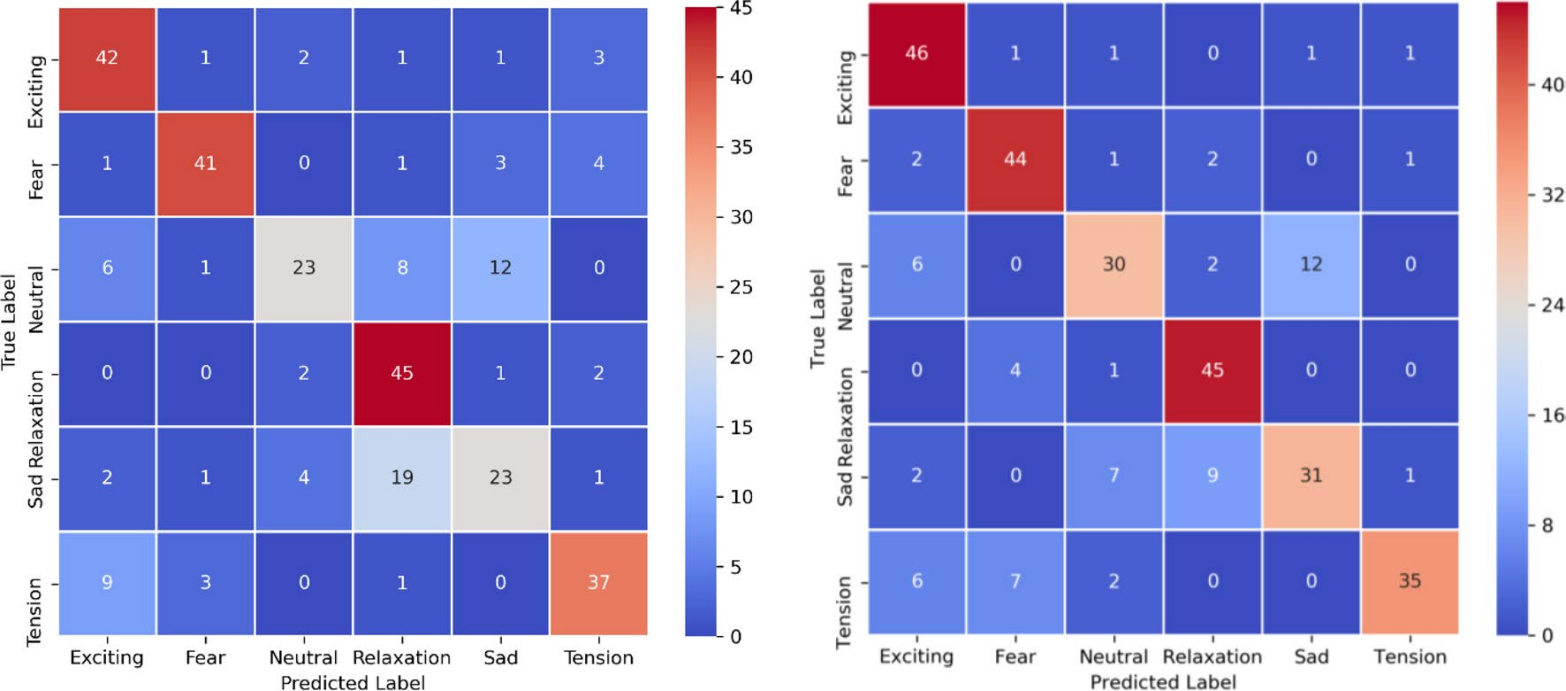
Confusion Metrix of the best performing multimodal using 50 test sample from each class. (Left) confusion matrix of music-video classifier and (right) confusion matrix of separable slow–fast network.

The statistical evaluation score was investigated using the confusion matrix and ROC curve. The confusion matrix visualizes the class-wise correlation of the classifier prediction. It counts the number of confused samples for each pair of classes. The ROC curve illustrates the performance of each classifier according to the various decision thresholds associated with it. The confusion matrix in Fig. 9 shows that the “Neutral” class is highly confusing to our classifier because it holds data that is similar to more than one class. The classifier result shows confusion on the samples from “Fear” and “Tension” classes because both classes hold similar music structure (mostly the rock and metal music). The “Sad” and “Relaxation” music videos have a similar silent nature so the classifier also confuses these classes. The “Excited” and “Fear” emotion classes were well classified compared to others because the body action and unique appearance captured by the video network play a vital role in it. Please refer the music video dataset description for the data characteristics within each class category. The evaluation results show that the separable slow–fast classifier boosted the overall performance and reduced the rate of confusion, as we expected. The best result was obtained when unsupervised features and MMTM blocks were used in the proposed networks.

**Figure 10:**
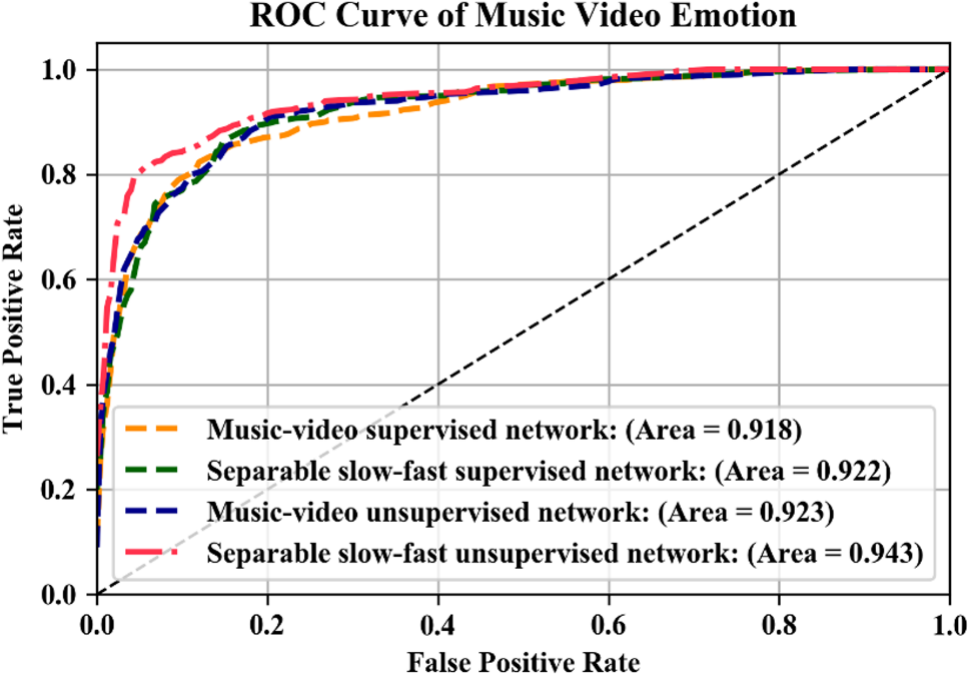
The ROC curve using several multimodal architectures trained on labelled and unlabeled dataset.

The ROC curve for the two multimodal architectures trained in the labelled and unlabeled dataset is shown in Fig. 10. The ROC curve for the unsupervised feature has a relatively high area under the curve (AUC) score compared to the fully supervised network. The separable slow–fast network trained on an unlabeled dataset performed more accurately, with fewer number of training parameters and the highest ROC-AUC score.

### Time synchronized emotion prediction

**Figure 11:**
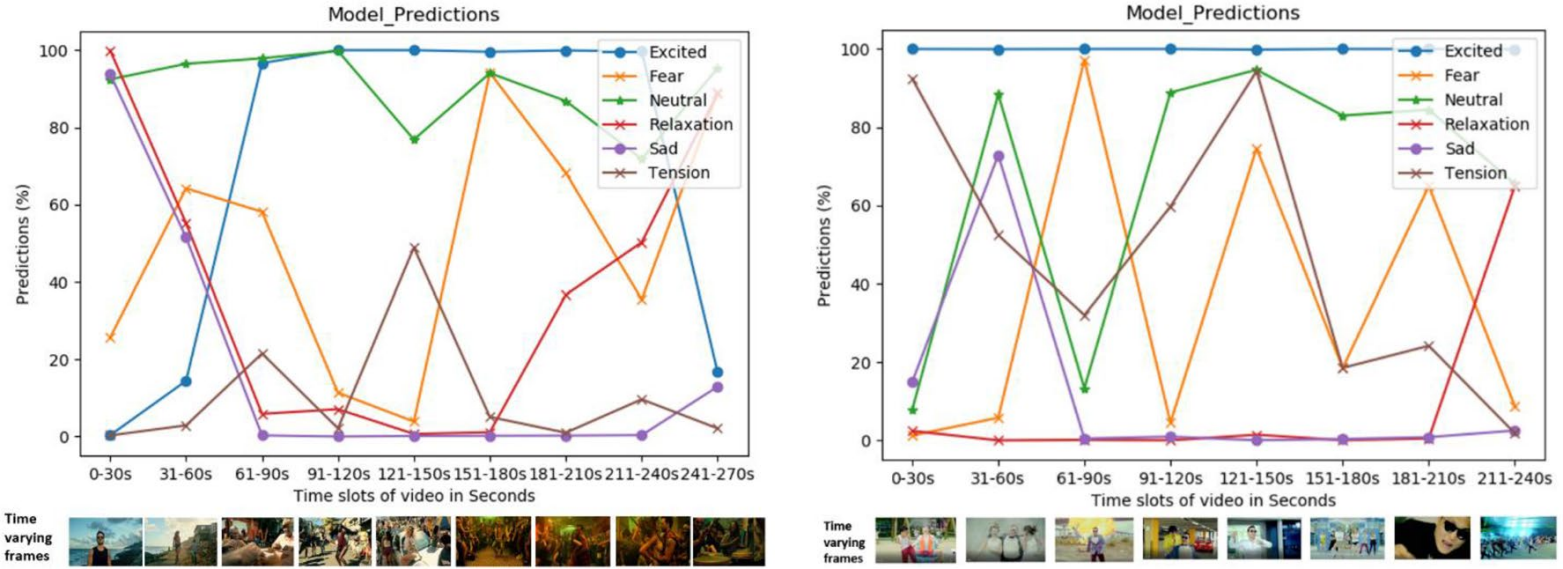
Time synchronized emotion prediction for specific music videos. (Left) prediction result on Luis_Fonsi and (right) prediction redult on Gangnam Style music video.

An emotional relationship between consumers and music videos has been shown in^74–76^ to be essential cross-culturally to promote a song or album. The number of views on a music video also support this claim. In this experiment, we evaluated the relationship of human emotion to music videos. We present the results of our best performing network on two music video with billions of views on YouTube at the time of writing. The prediction results of “Despacito” (https://www.youtube.com/watch?v=kJQP7kiw5Fk) (7.54B views at the time of writing) and “Gangnam Style” (https://www.youtube.com/watch?v=9bZkp7q19f0) (4.19B views at the time of writing) are shown in Fig. 11 on the left and right, respectively.

The prediction result (in percentages) for each class is illustrated with individual curves depicted across 30 s increments. We used the Sigmoid activation function to find the correlation of our six emotion categories. The highest activated class is illustrated with the highest score and the second or third most probable classes are further down on the vertical axis. One video frame is illustrated at the bottom according to the time for illustration, but the musical components are also responsible for the final decision.

### Comparisons with past studies

This research analyzed music video emotion using unsupervised and supervised methods. The dataset and methods are novel so an exact comparison with previous studies is not possible. The past studies are based on supervised method and there is no public dataset or method that treats the music video in an unsupervised manner, as far as we know.

The researches related to music emotion^8,14,77^ have presented varying evaluation scores for varying emotion class categories as shown in Table 3. One study of music video^46^ first proposed a Gaussian mixture model (GMM) using low-level music and video features. The dataset used in that research was limited to 120 samples with an 8-class category. Another study^47^ used audio and video features for music video emotion classification from a pre-trained network and an inbuilt library. The study used 18 class categories at a feature level and the dataset is not publicly available. A deep learning-based study (MM in Table 3)^5^ used 2D and 3D convolution methods for audio and video processing respectively and used transfer learning for music video emotion classification. The limitation of this study was the large complexity and that the test samples were taken from similare training samples. A recent study MVF^36^ use the same labelled dataset used in this study for music video affective computing using audio, video, and facial expression. This study shows the performance increment using multi-modal representation and illustrated the emotion classification score for each information source.

**Table 3:** Comparisons with past studies.

Method	Dataset	Data type	Emotion class	Score
RNN^14^	LastFM	Music	4	0.542 (Accuracy)
CNN^75^	CAL500 CAL500exp	Music	18	0.534 (F1-score)
		Music	18	0.709 (F1-Score)
SVM^15^	Own	Music	4	0.764(F1-Score)
GMM^46^	DEAP120	Music and video	8	0.90 (Accuracy)
CLR^47^	CAL500	Music and video	18	0.744 (Accuracy)
MM^5^	MVED_v1	Music and video	6	0.88 (F1-Score)
MVF^36^	MVED_v2	Music, video and face expression	6	0.73 (F1-Score)
Our (Supervised)	MVED_v2	Music and video	6	0.70 (F1-Score)
Our (Unsupervised)	MVED_v2	Music and video	6	0.77 (F1-Score)

In contrast to previous research, this research used unsupervised and supervised methods and datasets for music video emotion classification using end-to-end training. Our dataset is more diverse and improved in the sense that it can be used to train the deep neural network and is publicly available with the hope of attracting new researchers. Compared to past studies, this study proposed new methods and used various statistical and visual analyses to support the resulting scores. In Table 3, we quantitatively compare our current study with past related research. The proposed classifier cannot outperform some past results in terms of quantitative comparison, but they are qualitatively robust because the networks are trained on a relatively large data sample with two sources of inputs. Hence, the network capacity is more diverse and applicable for real-world applications. The visual analysis results support this claim.

## Conclusions

Music video affective computing is highly valuable for personal use to industrial application but little work has been done in this area in the past. The reason might be the lack of labeled dataset and annotation difficulties. Some research has focused on music emotion analysis; however, limited attention has been given to music videos, even though they are a widely used content on the Internet today. In this research, we present an unsupervised encoder-decoder paradigm for music video emotion representation. The unsupervised and supervised networks are compared with end-to-end training on a novel music video dataset. The visual and acoustic emotional clues of the music video are used in the multimodal representation with run time feature sharing and are boosted using a multimodal transfer module. The proposed multimodal architectures were first designed using general 2D/3D convolution and later the network complexity was drastically reduced by channel and filter separable convolution. We compared multiple unsupervised and supervised multimodal architectures using different evaluation metrics and visual analysis. The proposed method was found to be better in the qualitative analyses compared to past research.

There is great potential for future studies related to music videos. The robust network architecture, standard dataset, emotion representation framework, and new evaluation techniques are an important part of this research. Time synchronized emotion analysis and multimodal architecture using more information sources such as lyrics, user comments, and facial information can open new opportunities for this field. We have made our code and dataset publically available to attract new researchers and receive feedback for this study.

## Data Availability

The authors declare that all training and testing data and codes supporting this study are available from the first author upon reasonable request. All other data supporting this study are available within the article. The labelled dataset is available at https://drive.google.com/drive/u/6/folders/0AIVtKo7r0hTqUk9PVA and processed dataset is available at https://zenodo.org/record/4542796#.YCxqhWgzaUk.
